# Chlorogenic acid: a review on its mechanisms of anti-inflammation, disease treatment, and related delivery systems

**DOI:** 10.3389/fphar.2023.1218015

**Published:** 2023-09-13

**Authors:** Jianhuan Huang, Mingxiang Xie, Liang He, Xiaoping Song, Tianze Cao

**Affiliations:** ^1^ Breast Surgery, Central Laboratory, The Fifth Affiliated Hospital, Southern Medical University, Guangzhou, Guangdong, China; ^2^ The Graduate School, Guilin Medical University, Guilin, Guangxi, China; ^3^ Department of Anesthesiology, The Fifth Affiliated Hospital, Southern Medical University, Guangzhou, Guangdong, China

**Keywords:** chlorogenic acid, inflammatory response, signaling pathways, disease treatment, related delivery systems

## Abstract

Chlorogenic acid is a bioactive compound ubiquitously present in the natural realm, lauded for its salient anti-inflammatory and antioxidant attributes. It executes its anti-inflammatory function by moderating the synthesis and secretion of inflammatory mediators, namely, TNF-α, IL-1β, IL-6, IL-8, NO, and PGE2. Concurrently, it modulates key signaling pathways and associated factors, including NF-κB, MAPK, Nrf2, and others, bestowing protection upon cells and tissues against afflictions such as cardio-cerebrovascular and diabetes mellitus. Nevertheless, the inherent low bioavailability of chlorogenic acid poses challenges in practical deployments. To surmount this limitation, sophisticated delivery systems, encompassing liposomes, micelles, and nanoparticles, have been devised, accentuating their stability, release mechanisms, and bioactivity. Given its innate anti-inflammatory prowess and safety profile, chlorogenic acid stands as a promising contender for advanced biomedical investigations and translational clinical endeavors.

## 1 Introduction

Chlorogenic acid (CGA), alternatively termed as coffee tannic acid and coffee ellagic acid, is a water-soluble phenolic acid synthesized by plants during aerobic respiration (Liu et al., 2020a). This acid arises from the esterification of caffeic acid and quinic acid, boasting a molecular architecture characterized by three labile groups: ester bonds, unsaturated double bonds, and dibasic phenols (Yu et al., 2018). Key functional groups underpinning its bioactivity encompass hydroxyl, carboxyl, and o-diphenol hydroxyl groups (Wang. et al., 2017a). Empirical research has underscored the efficacy of chlorogenic acid in ameliorating inflammatory ailments, attributing this to its multifaceted targeting of inflammatory pathways and its diminished propensity for drug resistance (Wang et al., 2022a; Fumiere et al., 2022; Feng et al., 2023). Specifically, chlorogenic acid can attenuate cardiac and cerebral afflictions triggered by inflammation and oxidative stress, conferring cardiovascular and cerebrovascular protective effects (Wang et al., 2020a; Liu et al., 2022; Zheng et al., 2022). Concurrently, research has illuminated chlorogenic acid’s potential in enhancing metabolic parameters in hepatic and cardiac contexts, serving as a bulwark against obesity, cardiovascular maladies, and diabetes (Zhu et al., 2019; Zhang and Xu, 2022; Simona et al., 2023). In the realms of obesity and diabetes, chlorogenic acid has been identified to impede sugar absorption by human intestinal epithelial cells (Caco-2) *via* the modulation of Glut gene expression (Barreto et al., 2022). Additionally, it augments postprandial glycemic regulation, glucagon-like peptide-1 (GLP-1) response, and insulin sensitivity in normative populations (Aya et al., 2022; Zang et al., 2022). Given its distinctive anti-inflammatory attributes and pronounced benefits concerning cardiovascular, cerebrovascular, and diabetic conditions, chlorogenic acid is gaining traction in pharmaceutical and biomedical research arenas.

**FIGURE 1:**
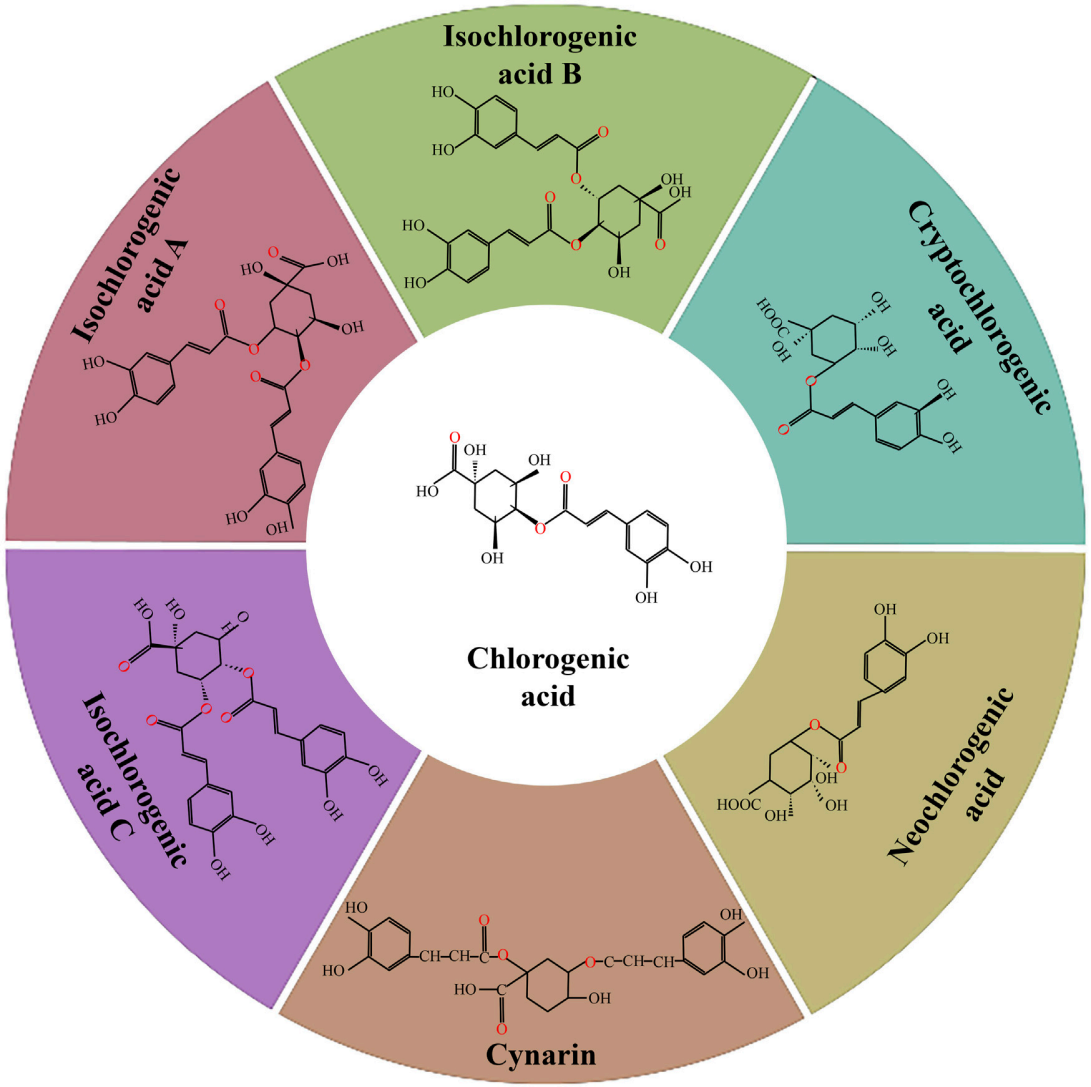
Molecular structure of chlorogenic acid and its isomers.

However, there are inherent challenges with chlorogenic acid, primarily its chemical instability, limited lipid solubility, suboptimal bioavailability, and vulnerability to esterase-mediated degradation (Jitka et al., 2004; Xiong et al., 2013). Serendipitously, chlorogenic acid undergoes isomerization during extraction, yielding more stable forms in nature, which effectively addresses some of these limitations (Dorrell, 1976). This isomerization engenders six principal isomers, notably cryptochlorogenic acid (4-caffeoylquinic acid), neochlorogenic acid (5-caffeoylquinic acid), and a series of isochlorogenic acids (Meinhart et al., 2019), as depicted in Figure 1. The isomerized chlorogenic acid retains its biologically active profile, exhibiting potent anti-inflammatory and antioxidant capacities (Song et al., 2023). To further refine the bioavailability of chlorogenic acid and circumvent its degradation, myriad packaging and delivery modalities have been innovated, such as liposomes, micelles, and nanoparticles, addressing the twin challenges of chemical instability and suboptimal bioavailability, thus broadening the horizon for its clinical deployment.

In this manuscript, a meticulous exploration of chlorogenic acid, its derivational isomers, and their associated biological mechanisms is undertaken. Furthermore, in anticipation of prospective practical challenges, drug delivery systems optimized for chlorogenic acid are elucidated.

## 2 Inhibition of inflammatory mediators by chlorogenic acid

At the molecular intersection of inflammatory pathways, chlorogenic acid predominantly impedes the synthesis and secretion of inflammatory mediators such as TNF-α, NO, COX-2, and PGE2. These mediators interplay in a complex manner, instrumental in the inception and progression of inflammation.

### 2.1 Inhibition of production and release of pro-inflammatory cytokines

Under conditions of excessive immunological perturbation, a protracted inflammatory response can be initiated, thereby inflicting collateral damage upon native cellular structures and instigating pathophysiological transformations (Qian et al., 2014). Notably, isochlorogenic acid has been identified to markedly attenuate pro-inflammatory cytokine levels, encompassing TNF-α, IL-6, IL-8, and IFN-γ, while concurrently enhancing anti-inflammatory cytokine concentrations, such as IL-10, ultimately mitigating inflammation in chicken fallopian tubes (Wu et al., 2022). In a landmark study, elevated concentrations of chlorogenic acid were found to shift macrophages away from classical inflammatory pathways, favoring their differentiation mediated by Coxsackie virus B3 (CVB3) towards the M2 pathway. This shift culminated in reduced synthesis of pro-inflammatory cytokines like TNF-α, IL-6, and IL-8 (Zhu et al., 2021). Analogously, chlorogenic acid demonstrated efficacy in curbing the proliferation of hemolytic *streptococcus*, attenuating the production of IL-6, TNF-α, and IL-1β, thus effectively quelling the inflammatory cascade in hemolytic streptococcus-infected mice (Hong et al., 2022). Subsequent animal studies have emphasized the robust antioxidant potential of chlorogenic acid, highlighting its ability to curtail serum TNF-α levels, neutralize oxygen radicals, enhance superoxide dismutase (SOD) concentrations, and foster the repair of collagen fibers, making it a promising candidate for oral ulcer therapy in murine models (Liu et al., 2020b; Tang et al., 2022). Furthermore, chlorogenic acid evidenced potential in diminishing the expression of pro-inflammatory markers like TNF-α and IL-6, thus counteracting the inflammatory sequelae in diabetic rodents induced by streptozotocin (Kang et al., 2011). Pertinently, reactive oxygen species (ROS) and reactive nitrogen species (RNS) have been earmarked as viable therapeutic agents for type 1 diabetes-induced inflammation (Kang et al., 2011). Additionally, research delineated the efficacy of chlorogenic acid in abating intestinal inflammation, fortifying the intestinal barrier, and ameliorating glucose metabolism dysregulation in type 2 diabetic rodents (Zhou et al., 2022).

### 2.2 Inhibition of NO production and release

Inducible Nitric Oxide Synthase (iNOS), predominantly located within macrophages and leukocytes, facilitates NO synthesis, integral to the inflammatory cascade (Anavi and Tirosh, 2020; Patrícia et al., 2022). In the milieu of inflammation, NO serves as an amplifier. The genesis and activation of iNOS in tissues by pro-inflammatory cytokines catalyze NO synthesis. Excessive NO accumulation exacerbates inflammatory pathways, intensifying the response (Han et al., 2017). Chlorogenic acid has been observed to diminish NO and IL-6 concentrations, mollify LPS-induced inflammatory onslaughts in murine peritoneal macrophages (RAW264.7), curtail peroxidase (MPO) expression, and elevate tissue superoxide dismutase (SOD) levels, thus showcasing promise as a potential therapeutic agent for ulcerative colitis in mice (Li et al., 2023). *In vitro* macrophage experiments post-LPS induction revealed that both chlorogenic acid and cryptochlorogenic acid notably suppressed iNOS expression and NO synthesis (Hui et al., 2021). Compounds such as chlorogenic acid, cryptochlorogenic acid, and 5-caffeoylquinic acid manifested inhibitory effects on NO secretion from LPS-stimulated murine macrophages (Song et al., 2015; Liu et al., 2019). *In vivo* experiments further highlighted the pronounced anti-inflammatory prowess of chlorogenic acid. Specifically, isochlorogenic acid C substantially reduced NO and PGE2 concentrations in the serum of mice subjected to carrageenan exposure, while the antioxidant properties of isochlorogenic acid A and isochlorogenic acid C neutralized free radical-induced damage and subdued inflammatory pathways in murine feet (Zhao et al., 2021). In a parallel vein, chlorogenic acid effectively stymied the acute inflammatory repercussions induced by xylene, leading to significant reductions in ear edema (Liu et al., 2019).

### 2.3 Inhibition of PGE2 production and release

Prostaglandin E2 (PGE2) plays a pivotal role in amplifying inflammatory reactions and augmenting nociceptive sensitivity. Concurrently, COX-2 is instrumental in enhancing PGE2 synthesis during inflammation (Choe et al., 2017). In instances of pronounced inflammatory perturbations, pro-inflammatory cytokines can instigate elevated COX-2 expression. This elevation, in turn, accelerates the metabolism of arachidonic acid (AA) to yield heightened levels of PGE2, perpetuating a positive feedback loop that intensifies the inflammatory cascade (Kang et al., 2011; Kang et al., 2017). Chlorogenic acid demonstrates its anti-inflammatory prowess by attenuating the M1 macrophage-mediated inflammatory response, achieved through the downregulation of genes pivotal to polarization, encompassing COX-2, IL-6, and TNF-α. As a corollary, the synthesis of PGE2 witnesses a concomitant reduction (Lan et al., 2021; Zhao, 2021). Furthermore, chlorogenic acid has exhibited efficacy in suppressing COX-2 expression in the context of acute pulmonary tissue injury in murine models (Kang et al., 2017).

## 3 Regulation of cellular signaling pathways by chlorogenic acid

### 3.1 Inhibition of NF-κB signaling pathway

The NF-κB complex primarily consists of two heterodimeric subunits, P50 and P65. In its quiescent state, NF-κB is sequestered in the cytoplasm, bound to IκB (an inhibitor of NF-κB). Upon activation, IκB undergoes phosphorylation and ubiquitination, facilitating the nuclear translocation of NF-κB to bind target genes, orchestrating the transcriptional modulation of the inflammatory cascade (Liu and Yao, 2005). Chlorogenic acid’s capacity to mitigate inflammation via impeding this signaling conduit has been substantiated across diverse cellular and tissue frameworks (Chen et al., 2021; Duan et al., 2022; Ji et al., 2022; Zhang and Xu, 2022). For instance, investigations have discerned that chlorogenic acid attenuates the inflammatory milieu in rheumatoid arthritis by suppressing NF-κB expression and its consequent DNA-binding activity, culminating in the inhibition of downstream TNF-α secretion (Fu et al., 2019). Several empirical assessments corroborate that chlorogenic acid, in a dose-responsive manner, downregulates inflammatory mediators including IL-6, TNF-α, IL-1β, and TLR2 by hindering the phosphorylation of NF-κB pathway proteins, positioning chlorogenic acid as a prospective therapeutic agent for dairy mastitis (Xu et al., 2023). Notably, one research endeavor illuminated chlorogenic acid’s potential to counteract the P65 upregulation, thus dampening the inflammatory perturbations in Caco-2 cells incited by interferon-gamma (IFNγ) and myristate (PMA) (Liang and Kitts, 2018). Moreover, in the context of LPS-induced retinal inflammation, chlorogenic acid curbed diabetic retinopathy by diminishing p65 phosphorylation and curtailing the release of IL-6 and TNF-α(Xian et al., 2019).

Further delving into the mechanistic intricacies, there’s burgeoning evidence elucidating the upstream regulatory axes through which chlorogenic acid navigates the NF-κB signaling trajectory (Zhao et al., 2020). Pioneering studies have demystified chlorogenic acid’s competency in negating the TLR4/NF-κB signaling cascade, leading to a conspicuous reduction in TLR4, MyD88, and NF-κB expression levels, thereby ameliorating oxidative impairments in small intestinal tissue and refining the inflammatory demeanor of septic rat epithelial cells (Wang et al., 2023a). The TOLL receptor 4 (TLR4)/MyD88 axis might further invigorate IKK, engendering IκB degradation and triggering NF-κB activation, thereby catalyzing the inflammatory cascade (Matthias et al., 2009). Chlorogenic acid, by moderating the TLR4 pathway, can stymie the transcriptional vigor of NF-κB, offering a dual benefit of thwarting macrophage-derived foam cell proliferation and fostering their apoptosis, bestowing considerable cardiorenal protective attributes (Lin, 2019; Zhang and Xu, 2022).

In synthesis, chlorogenic acid adeptly impedes the NF-κB signaling cascade’s activation, primarily by stalling the phosphorylation events associated with IKK, IκB, and P65. Concurrently, it also suppresses the upstream signaling pathways, notably the TOLL receptor4 (TLR4)/MyD88 nexus, thereby manifesting robust anti-inflammatory attributes.

### 3.2 Inhibition of MAPK signaling pathway

MAPK represents a distinct category of serine/threonine kinases. The intricate MAPK signaling cascade encompasses three predominant pathways: ERK, P38, and JNK, each capable of transducing diverse signals to the nucleus, subsequently binding to specific proteins to initiate and amplify inflammatory responses.

Chlorogenic acid has been evidenced to quell the phosphorylation of the trio of kinases–ERK1/2, P38, and JNK, subsequently mitigating dextrose sulfate (DSS)-provoked intestinal inflammation and oxidative stress (Gao et al., 2019). In the context of LPS-mediated cardiomyocyte inflammation in mice, chlorogenic acid has been shown to attenuate myocardial inflammation, chiefly by obviating upstream MEK phosphorylation and suppressing ERK expression (Geng et al., 2019a). Moreover, chlorogenic acid conferred protection against myocardial ischemia-reperfusion-induced inflammatory lesions and oxidative distress in rats, achieved by thwarting the activation of the MEK/ERK signaling axis and diminishing the myocardial infarct dimensions (Geng et al., 2019a; Geng et al., 2019b; Geng et al., 2019b). Furthermore, chlorogenic acid inhibited P38 phosphorylation, culminating in a diminished release of downstream pro-inflammatory mediators IL-6 and TNF-α, as evidenced in a model involving transforming growth factor (TGF)-β1-induced hepatic stellate cell (HSC) activation (Wang et al., 2017b). By constraining p38 phosphorylation, chlorogenic acid fostered autophagic processes in *Salmonella typhi* (ST)-infected mouse intestinal epithelial cells, leading to a reduction in mouse mortality (Tan et al., 2020). In a model of cardiac injury using TNF-α-induced human induced pluripotent stem cell-derived cardiomyocytes (hiPSC-CMs), chlorogenic acid, upon pre-administration, counteracted TNF-α-mediated cellular detriment *via* a mechanism potentially linked to the inhibition of the JNK pathway, and by extension, the overarching MAPK signaling conduit (Tian et al., 2019).

### 3.3 Activation of the Nrf2 signaling pathway

Nrf2, a nuclear transcription factor E2-related factor, plays an instrumental role in combating oxidative stress and dampening inflammatory cascades. Chlorogenic acid is renowned for its anti-inflammatory and antioxidant prowess, largely attributed to its capacity to activate the Nrf2 signaling paradigm. Once actuated, Nrf2 translocates to the nucleus, binding to its target genes and orchestrating the transcription of a cadre of affiliated proteins, including hemoglobin oxidase-1 (HO-1), pro-quinone oxidoreductase-1 (NQO-1), and γ-glutamylcysteine synthetase (γ-GCS), which collectively render anti-inflammatory and antioxidant effects.

Research indicates that chlorogenic acid facilitates the liberation of Nrf2 from its binding partner keap1, culminating in Nrf2 activation and concomitant upregulation of HO-1 and NQO-1 (Gao et al., 2021). A seminal study posited that chlorogenic acid mitigated acetaminophen (APAP)-induced inflammatory perturbations in liver tissues, a modulation attributable to Nrf2 activation; intriguingly, this anti-inflammatory efficacy of chlorogenic acid was notably compromised in the absence of Nrf2 (Hu et al., 2020). In a rat model simulating sepsis, chlorogenic acid heightened Nrf2 protein expression, resulting in the augmented release of HO-1 and NQO1, thereby curbing macrophage activity and ameliorating both inflammation and oxidative stress-induced pulmonary tissue damage (Wang et al., 2020b). Moreover, chlorogenic acid displayed a remedial effect against LPS-triggered acute kidney injury by abrogating inflammatory responses and systemic oxidative stress, a mechanism presumably linked to the activation of the Nrf2 pathway, bolstering Nrf2 nuclear translocation and subsequent protein expression of downstream targets HO-1 and NQO1 (Feng et al., 2022). Studies have also demonstrated that chlorogenic acid counteracts type 1 diabetic retinopathy (DR) manifestations by fortifying Nrf2 activation, obstructing TNF-α-mediated retinal endothelial-mesenchymal (EndoMT) and epithelial-mesenchymal (EMT) transitions, and mitigating oxidative damage (Hao et al., 2022). Furthermore, chlorogenic acid manifests a robust protective effect against Doxorubicin (DOX)-mediated cardiotoxicity in rats; this cardioprotection appears to be hinged on chlorogenic acid’s ability to suppress apoptotic marker caspase-3 expression and tyrosine, whilst enhancing Nrf2 and Ho-1 expression in the cardiac milieu (Betul et al., 2023).

## 4 Chlorogenic acid in disease treatment

Chlorogenic acid, recognized as a pivotal natural compound, possesses a diverse array of pharmacological properties. Particularly noteworthy are its anti-inflammatory and antioxidant capabilities, which render it efficacious in the prophylaxis and therapeutic management of cardiovascular and cerebrovascular maladies.

### 4.1 The therapeutic effect of chlorogenic acid on cardiovascular diseases

Empirical research underscores that chlorogenic acid mitigates the severity of ventricular myocardial infarction and augments cardiac functionality by attenuating inflammatory mediators and enhancing the activity of antioxidant enzymes, thereby serving as an instrumental player in forestalling myocardial infarction (Wang et al., 2020a). Additionally, chlorogenic acid presents a prophylactic approach against thrombotic events following valve replacement in advanced heart valvular disorders, attributable to its anticoagulant properties and its role in fostering endothelial cell proliferation (Chen et al., 2023).

Concomitantly, its cardioprotective attributes are further elucidated through modulating pertinent signaling pathways. Chlorogenic acid has been documented to counteract doxorubicin-triggered cardiotoxicity by orchestrating the Nrf2/HO-1 signaling cascade, thus manifesting profound cardioprotective ramifications (Betul et al., 2023). Furthermore, chlorogenic acid impedes the progression of cardiac fibroblasts (CFs), a phenomenon potentially steered by the TGF-βl/Smads signaling pathway (Niu et al., 2022).

### 4.2 The therapeutic effect of chlorogenic acid on cerebrovascular diseases

Chlorogenic acid offers protection to hippocampal neurons, primarily by negating mitochondrial aberrations and calcium overload, positioning it as a promising therapeutic candidate for epilepsy (Zhang et al., 2023a). Apoptotic pathways largely underpin neuronal impairment following cerebral ischemia-reperfusion (I/R) insult. In this context, chlorogenic acid markedly diminishes the expression of the NF-κB protein in hippocampal neurons of I/R-challenged rats, unveiling a novel therapeutic target for ischemic cerebrovascular accidents (Shiva et al., 2022). Chlorogenic acid, when administered prophylactically, ameliorates cognitive impairments, unveiling neuroprotective attributes in transient forebrain ischemia (TFI) scenarios. This neuroprotection appears to correlate with an upregulation in SOD2 expression facilitated by chlorogenic acid (Lee et al., 2020). Concurrently, chlorogenic acid elevates antioxidant enzyme expression and anti-inflammatory cytokine levels, while diminishing oxidative stress markers and pro-inflammatory cytokines (Lee et al., 2020). Additionally, the compound bolsters SOD activity, elevates GSH concentrations, curtails ROS and LDH production, reduces MDA accumulation, and ameliorates cerebral ischemia-reperfusion (CI/R) injury sequels (Liu et al., 2020).

### 4.3 The therapeutic effect of chlorogenic acid on diabetes

In streptozotocin (STZ)-elicited diabetic rat prototypes, chlorogenic acid demonstrates therapeutic potential against type 1 diabetes-mediated inflammation, achieved through the downregulation of IL-6 and tumor necrosis factor in rat circulatory systems (Lee et al., 2022). Beyond this, chlorogenic acid wields significance in addressing diabetes-associated complications. Diabetic hyperglycemic states activate the NF-κB signaling pathway in neuronal structures, culminating in diabetic encephalopathy and subsequent cognitive decline (F et al., 2023). Chlorogenic acid ameliorates such cognitive deficits, a process conceivably attributed to its inhibitory action on NF-κB and IL-6 within frontal brain structures (F et al., 2023). Excessive glucose levels can instigate endothelial cell dysfunction (Wang et al., 2021). In a diabetic murine model with aortic endothelium-dependent relaxation under high glucose challenge, chlorogenic acid mitigates endothelial anomalies through the activation of the Nrf2 antioxidant pathway (Wang et al., 2021).

## 5 The clinical trials of chlorogenic acid

In the burgeoning realm of chlorogenic acid research, clinical trials have illuminated promising results. Observational studies discern that chlorogenic acid precipitates a notable decrement in C-reactive protein (CRP) and IL-6 concentrations in subjects, augments cardiometabolic indices, and exhibits a commendable therapeutic efficacy in cardiovascular disorders (Asieh et al., 2021). Furthermore, chlorogenic acid has been credited with attenuating body mass in diabetic cohorts *via* the amplification of intestinal bifidobacterial populations (Asieh et al., 2020), and has manifested improvement in endothelial dynamics among stage 1 hypertensive patients (Masato et al., 2019). In summation, the paramountcy of chlorogenic acid in disease prophylaxis and management cannot be overstated, and ensuing research endeavors will expound upon clinical trials to discern the intricate mechanisms mediated by chlorogenic acid.

## 6 Chlorogenic acid-loaded delivery systems

While chlorogenic acid holds promise as a therapeutic agent in mitigating inflammatory disorders, cardiovascular and cerebrovascular maladies, and diabetes, the intrinsic limitations posed by its molecular configuration, marked by unstable moieties, fickle chemical traits, suboptimal lipid solubility, and diminished bioavailability *in vivo*, constrain its biomedical potential. Albeit various chlorogenic acid isomers exhibit salient advantages over their native counterpart, they occasionally betray certain toxicological concerns and adverse manifestations. Consequently, a panoply of chlorogenic acid encapsulation and delivery stratagems have been envisaged, encompassing liposomes, micelles, and nanoparticles, among others (Li et al., 2021; Li et al., 2022).

### 6.1 Liposomes

Endowed with a molecular architecture chiefly composed of phospholipids and fatty acids, and reminiscent of cellular membranes, liposomes have been canonized for their innate biocompatibility and biodegradability (Wang et al., 2023b). These entities, comprising amphiphilic molecules orchestrating into bilayered vesicles, emerge as exemplary conduits for an assortment of molecular species with varying polarities (E et al., 2021). A salient advantage of liposomes resides in their ability to stabilize active agents, surmount barriers thwarting cellular drug uptake, extend the retention span of therapeutics within target cells, and thereby potentiate therapeutic outcomes (Tapan et al., 2016). An *in vivo* evaluation heralded a 1.29-fold enhancement in the bioavailability of a chlorogenic acid extract upon its encapsulation within soy phosphatidylcholine and cholesterol liposomes (maintaining a liposome-to-chlorogenic acid ratio of 6:1), suggesting liposomal encapsulation as a viable strategy for bolstering compound bioavailability (Feng et al., 2016). In the milieu of simulated *in vitro* gastrointestinal digestion (GID), a 40% decrement in chlorogenic acid content has been reported. However, juxtaposed against this observation, the chlorogenic acid content ensconced within liposomes remains predominantly conserved throughout gastrointestinal digestive episodes, forestalling its degradation or morphological transformations (Ailén et al., 2022).

### 6.2 Micelles

Distinguished from liposomes in their architectural nuances, micelles present themselves as closed lipid monolayers, manifesting with polar head groups on the exterior and hydrophobic tails sequestered internally. This unique configuration augments the solubility of an array of hydrophobic pharmaceuticals (Wang et al., 2023b). Furthermore, micelles amplify the permeability of drugs and enhance their effective concentration *in situ*, thereby augmenting the bioavailability of the encapsulated compound (Sabna et al., 2022). In a seminal study, chlorogenic acid was encapsulated onto poly (D, L-lactide-co-glycolide) (PLGA) and subsequently adorned with polyvinylpyrrolidone (PV) to fabricate nano-micelles. Subsequent *in vivo* mouse assays and *in vitro* assessments divulged that these nanomicelles not only elongate the tissue residence time but also modulate the sustained release of chlorogenic acid, underpinning their commendable biocompatibility (Li et al., 2022). In a different vein, micelles have found applicability in extraction protocols. Evidence suggests that the yield from ultrasonic-assisted micellar-mediated extraction (UAMME) trumps that from conventional aqueous extractions, whilst preserving the biological fidelity of the extract (Karolina et al., 2016).

### 6.3 Nanoparticles

Nanoparticles amplify the stability of bioactive moieties, facilitating their traverse across cellular membranes and biological impediments, thereby ensuring a controlled release within the target cellular milieu (Yao et al., 2017). One pivotal investigation showcased that bovine serum albumin (BSA)-facilitated chlorogenic acid silver nanoparticles (AgNPs-CGA-BSA) exude substantial antioxidant and anti-neoplastic properties across *in vivo* and *in vitro* matrices. Moreover, indices such as reduced glutathione (GSH), superoxide dismutase (SOD), and catalase (CAT) exhibited amplified activities in DLA cells and murine serum post AgNPs-CGA-BSA treatment, positing a prospective therapeutic avenue for T-cell lymphoma (Tamanna et al., 2022). Additionally, chlorogenic acid-encased mesoporous silica entities, tailored with hexahistidine peptide sequences, demonstrated a potential to attenuate metal-induced dermal inflammation in mice *via* robust nickel chelation. Such modifications resulted in a formidable 63.22% release of chlorogenic acid at the 10-h mark (Wang et al., 2022b).

## 7 Summary and discussion

In contemporary years, the exploration into chlorogenic acid’s anti-inflammatory mechanisms has burgeoned, with revelations permeating diverse scientific domains. At the molecular crossroads of inflammation, chlorogenic acid predominantly forestalls the synthesis and subsequent discharge of bioactive molecules such as TNF-α, NO, COX-2, and PGE2. Notably, it curtails the release of TNF-α and interleukin derivatives by thwarting the activation of both macrophages and monocytes. Analogously, it counteracts inflammation propagation by inhibiting nitric oxide synthesis. In circumstances where heightened inflammatory reactions elevate COX-2 expression, chlorogenic acid intervenes by attenuating PGE2 synthesis, thereby obviating inflammatory-induced tissue detriment. On the signalling axis, chlorogenic acid orchestrates a triad of regulatory modalities that modulate inflammatory pathway constituents. This, in turn, precludes exacerbations incited by primordial inflammatory agents or exogenous triggers, ensuring inflammation’s containment and resolution.

**FIGURE 2:**
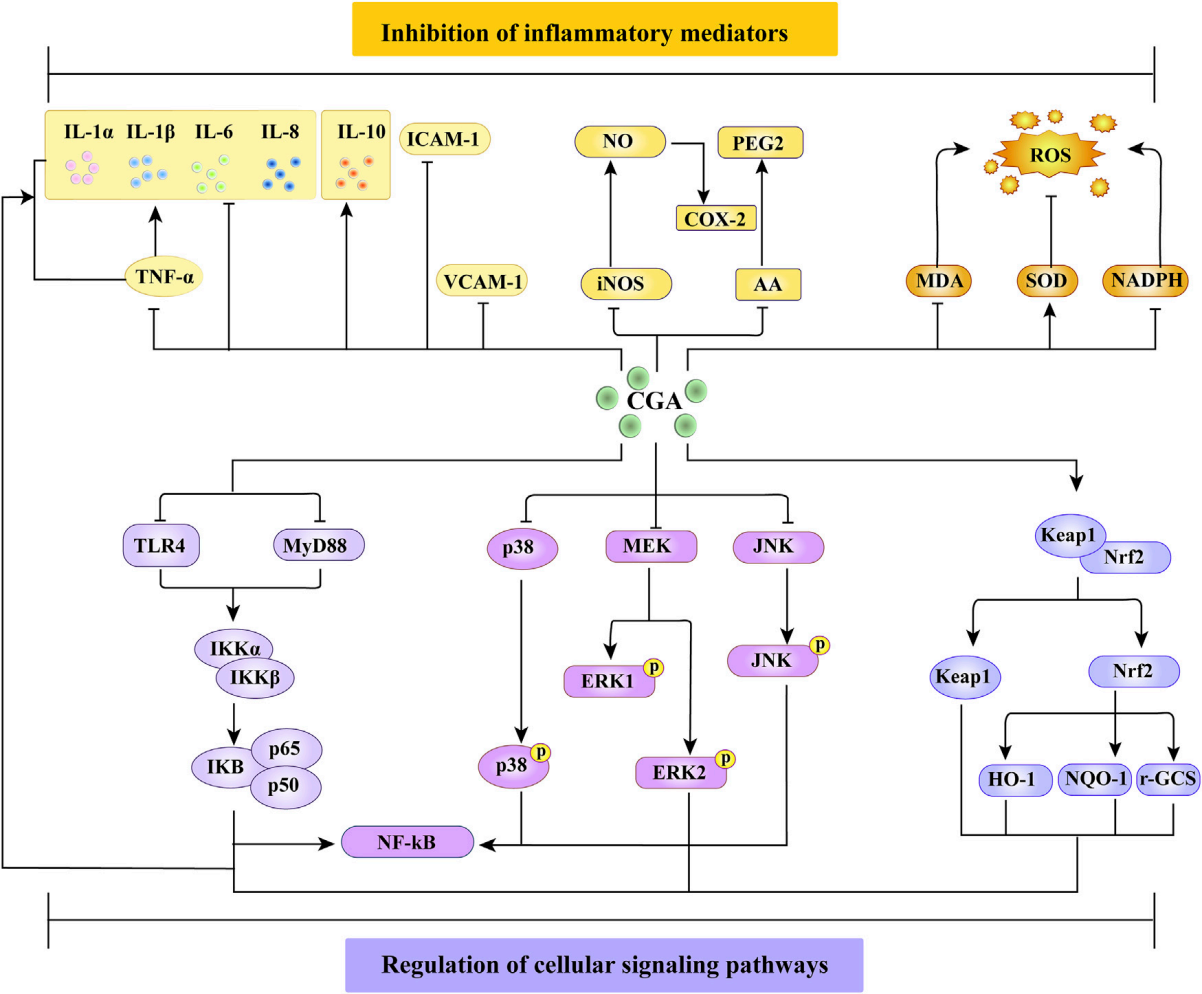
Anti-inflammatory mechanism of chlorogenic acid compounds.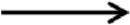:promote; 
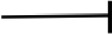
:inhibit; CGA:chlorogenic acid; VCAM-1:vascular cell adhesion molecule 1; MDA:malonaldehyde; NADPH:nicotinamide adenine dinucleotide phosphate; ROS:reactive oxygen species; TLR4:toll like receptor 4; MyD88:myeloid differentiation primary response gene (88); P-P38:phosphorylated P38 kinase; P-PERK1:phosphorylated extracellular signal-regulated protein kinase-1; P-PERK2:phosphorylated extracellular signal-regulated protein kinase-2; p-JNK:phosphorylated c-Jun amino-terminal kinase.

Chlorogenic acid exerts its anti-inflammatory prowess through a two-pronged molecular strategy. Primarily, it impedes the activation of NF-κB, p65, IκB, and IKK, ensuring that NF-κB is barred from nuclear translocation and subsequent target gene binding, thereby manifesting its anti-inflammatory attributes. Subsequently, chlorogenic acid stymies the phosphorylation of ERK1/2, JNK, and P38, effectively curtailing the MAPK signaling pathway. Furthermore, by fostering the expression of HO-1 and NOQ-1 and concurrently suppressing the secretion of pivotal inflammatory mediators such as IL-1β, IL-6, and IL-8 from macrophages, chlorogenic acid achieves this *via* the activation of Nrf2. The encapsulated anti-inflammatory mechanism of chlorogenic acid is depicted in Figure 2.

Additionally, chlorogenic acid holds promise in mitigating inflammatory disorders, cardiovascular anomalies, cerebrovascular conditions, diabetes, and an array of other ailments. This is achieved predominantly through the attenuation of inflammatory cytokine genesis and the fine-tuning of inflammation-centric signaling cascades. Notwithstanding its merits, the innate low bioavailability of chlorogenic acid presents a therapeutic conundrum. To this end, this manuscript elucidates several delivery modalities, including liposomes, micelles, and nanoparticles, envisaged to ameliorate the therapeutic limitations of chlorogenic acid. These delivery systems bolster the stability and bioactivity of chlorogenic acid, prolong its pharmacodynamic span, and enhance its bioavailability.

The current scientific literature offers a nascent understanding of the therapeutic efficacy and mechanistic intricacies of chlorogenic acid. While its profusion of anti-inflammatory targets, robust bioactivity, and ubiquitous presence in nature underscore its potential, several pivotal aspects warrant rigorous exploration. Notably, the majority of studies corroborating the bioactivity of chlorogenic acid are relegated to cellular and animal models; hence, the pressing need for expansive clinical trials to elucidate its therapeutic nuances further. Additionally, a discernible research void exists concerning the influence of physicochemical attributes on the delivery systems of chlorogenic acid, an avenue that necessitates rigorous academic attention to harness the full therapeutic potential of chlorogenic acid. Addressing these lacunae will pave the way for optimizing the anti-inflammatory, antioxidative, and potentially antineoplastic applications of chlorogenic acid. In summation, the research trajectory of chlorogenic acid is still in its incipient stages, and a plethora of investigative avenues beckons.

## References

[B1] Ailén A. Daniel M. Fernández d. P. P. Carmen G. M. d. Pilar M. (2022). Anti-inflammatory properties, bioaccessibility and intestinal absorption of sea fennel (crithmum maritimum) extract encapsulated in soy phosphatidylcholine liposomes. Nutrients 14 (1), 210. 10.3390/nu14010210 35011085 PMC8747172

[B2] Anavi S. Tirosh O. (2020). iNOS as a metabolic enzyme under stress conditions. Free Radic. Biol. Med. 146 (C), 16–35. 10.1016/j.freeradbiomed.2019.10.411 31672462

[B3] Asieh M. Reza M.-T. M. Sara K. Milad S. Hossein P. Samaneh E. (2020). Short term effects of coffee components consumption on gut microbiota in patients with non-alcoholic fatty liver and diabetes: A pilot randomized placebo-controlled, clinical trial. EXCLI J. 19, 241–250. 10.17179/excli2019-2021 32256270 PMC7105939

[B4] Asieh M. Reza M. T. M. Majid S. Mostafa Q. Shahin M. Hossein A. (2021). Effects of supplementation with main coffee components including caffeine and/or chlorogenic acid on hepatic, metabolic, and inflammatory indices in patients with non-alcoholic fatty liver disease and type 2 diabetes: A randomized, double-blind, placebo-controlled, clinical trial. Nutr. J. 20 (1), 35. 10.1186/s12937-021-00694-5 33838673 PMC8037901

[B5] Aya Y. Yuji M. Tohru Y. Masanobu H. Shigeru K. Noriko O. (2022). Effects of ingesting both catechins and chlorogenic acids on glucose, incretin, and insulin sensitivity in healthy men: A randomized, double-blinded, placebo-controlled crossover trial. Nutrients 14 (23), 5063. 10.3390/nu14235063 36501092 PMC9737369

[B6] Barreto P. J. A. Nelson A. Susana M. Fátima M. Oliveira M. B. P. P. Martel F. (2022). Green/roasted coffee and silverskin extracts inhibit sugar absorption by human intestinal epithelial (Caco-2) cells by decreasing GLUT2 gene expression. Foods 11 (23), 3902. 10.3390/foods11233902 36496710 PMC9737879

[B7] Betul C. Ahmet H. Yesim Y. Betul D. Mustafa O. Behzad M. (2023). Chlorogenic acid attenuates doxorubicin-induced oxidative stress and markers of apoptosis in cardiomyocytes via Nrf2/HO-1 and dityrosine signaling. J. personalized Med. 13 (4), 649. 10.3390/jpm13040649 PMC10140899 37109035

[B8] Chen J. Luo Y. Li Y. Chen D. Yu B. He J. (2021). Chlorogenic acid attenuates oxidative stress-induced intestinal epithelium injury by Co-regulating the PI3K/akt and IκBα/NF-κB signaling. Antioxidants 10 (12), 1915. 10.3390/antiox10121915 34943017 PMC8750628

[B10] Chen X. Yu T. Kong Q. Xu H. Zhao Z. Li G. (2023). A chlorogenic acid functional strategy of anti-inflammation, anti-coagulation and promoted endothelial proliferation for bioprosthetic artificial heart valves. J. Mater. Chem. 11, 2663–2673. 10.1039/d2tb02407a 36883900

[B11] Choe J. Yoon Y. Kim J. Jung Y. (2017). Positive feedback effect of PGE2 on cyclooxygenase-2 expression is mediated by inhibition of Akt phosphorylation in human follicular dendritic cell-like cells. Mol. Immunol. 87, 60–66. 10.1016/j.molimm.2017.04.004 28407559

[B12] Danielle E. L. Rudolf G. A. Elizabeth A. F. Debra T. A. (2021). Liposome composition in drug delivery design, synthesis, characterization, and clinical application. Adv. drug Deliv. Rev. 176, 113851. 10.1016/j.addr.2021.113851 34224787

[B13] Dorrell D. G. (1976). Chlorogenic acid content of meal from cultivated and wild Sunflowers1. Crop Sci. 16 (3), 422–424. 10.2135/cropsci1976.0011183x001600030028x

[B14] Duan S. Lu W. Yu W. Zuo S. (2022). Chlorogenic acid reducing LPS-induced neuroinflammatory iniury in microglia by inhibiting NF-κB/NL-RP3 inflammasome pathway. China Pharm. 25 (5), 758–764. 10.19962/j.cnki.issn1008-049X.2022.05.002

[B15] Feng S. Zhang Y. Fu S. Li Z. Zhang J. Xu Y. (2023). Application of Chlorogenic acid as a substitute for antibiotics in Multidrug-resistant Escherichia coli-induced mastitis. Int. Immunopharmacol. 114, 109536. 10.1016/j.intimp.2022.109536 36700763

[B16] Feng X. Xin X. Huang J. Zhao Y. Liu J. Tang Q. (2022). Protective effect of chlorogenic acid on sepsis-induced acute kidney injury in rats. Chin. J. Veterinary Med. 58 (5), 64–70+62+133.

[B17] Feng Y. Sun C. Yuan Y. Zhu Y. Wan J. Firempong C. K. (2016). Enhanced oral bioavailability and *in vivo* antioxidant activity of chlorogenic acid via liposomal formulation. Int. J. Pharm. 501 (1-2), 342–349. 10.1016/j.ijpharm.2016.01.081 26861689

[B18] Fu X. Lyu X. Liu H. Zhong D. Xu Z. He F. (2019). Chlorogenic acid inhibits BAFF expression in collagen-induced arthritis and human synoviocyte MH7A cells by modulating the activation of the NF-κB signaling pathway. J. Immunol. Res. 2019, 8042097. 10.1155/2019/8042097 31240234 PMC6556285

[B19] Fumiere L. M. Nathacha d. A. S. Merigueti d. S. C. M. Karla L. Almeida d. F. A. F. Poltronieri P. H. (2022). Chlorogenic acid and caffeine contents and anti-inflammatory and antioxidant activities of green beans of conilon and arabica coffees harvested with different degrees of maturation. J. Saudi Chem. Soc. 26 (3), 101467. 10.1016/j.jscs.2022.101467

[B20] Gao F. Fu K. Li H. Feng Y. Tian W. Cao R. (2021). Chlorogenic acid ameliorates mice clinical endometritis by activating Keap1/Nrf2 and inhibiting NFκB signalling pathway. J. Pharm. Pharmacol. 73 (6), 785–795. 10.1093/jpp/rgab020 33734387

[B21] Gao W. Wang C. Yu L. Sheng T. Wu Z. Wang X. (2019). Chlorogenic acid attenuates dextran sodium sulfate-induced ulcerative colitis in mice through MAPK/ERK/JNK pathway. BioMed Res. Int. 2019, 6769789. 10.1155/2019/6769789 31139644 PMC6500688

[B22] Geng J. Zhang Y. Li W. (2019a). Effects of chlorogenic acid on MEK/ERK signaling pathway and inflammatory reaction in cardiomyocyte. West. J. Traditional Chin. Med. 32 (11), 36–39.

[B23] Geng J. Zhang Z. Li W. (2019b). Protective effect of chlorogenic acid preconditioning on myocardial ischemia-reperfusion injury in rats. Chin. J. Mod. Appl. Pharm. 36 (6), 682–685. 10.13748/j.cnki.issn1007-7693.2019.06.007

[B24] Han Y. Zhang Y. Lai X. Liu H. Kou J. (2017). Advances of regulatory effects of traditional Chinese medicine on NOS/NO system. J. China Pharm. Univ. 48 (1), 8–15.

[B25] Hao O. Ao D. Zhou L. Zhang T. Lu B. Wang Z. (2022). Chlorogenic acid improves diabetic retinopathy by alleviating blood-retinal-barrier dysfunction via inducing Nrf2 activation. Phytotherapy Res. PTR 36 (3), 1386–1401. 10.1002/ptr.7401 35133045

[B26] Hong L. Liu W. Ying H. Peng K. Chen C. Shi H. (2022). Effects of chlorogenic acid extracted from honeysuckle on coagulation and inflammation related indexes in Streptococcus infected mouse model. Biol. Chem. Eng. 8 (6), 72–74.

[B27] Hu F. Guo Q. Wei M. Huang Z. Shi L. Sheng Y. (2020). Chlorogenic acid alleviates acetaminophen-induced liver injury in mice via regulating Nrf2-mediated HSP60-initiated liver inflammation. Eur. J. Pharmacol. 883(prepublish). 173286, 10.1016/j.ejphar.2020.173286 32603696

[B28] Hui H. Chen Q. Tian G. Shan Q. Qin L. (2021). Study on anti-inflammatory quality markers of Euodia rutaecarpa based on spectrum-efficacy correlation analysis. Chin. Traditional Herb. Drugs 52 (9), 2589–2596.

[B29] Ji Q. Zhang M. Wang Y. Chen Y. Wang L. Lu X. (2022). Protective effects of chlorogenic acid on inflammatory responses induced by *Staphylococcus aureus* and milk protein synthesis in bovine mammary epithelial cells. Microb. Pathog. 171, 105726. 10.1016/j.micpath.2022.105726 35995255

[B30] Jitka P. Sárka C. Petra M. Jan H. Vilím S. (2004). Chemoprotective effect of plant phenolics against anthracycline-induced toxicity on rat cardiomyocytes. Part III. Apigenin, baicalelin, kaempherol, luteolin and quercetin. Phytotherapy Res. PTR 18 (7), 516–521. 10.1002/ptr.1462 15305308

[B31] Kang J. Zhang L. Liu X. Yin Z. (2017). Effect of chlorogenic acid on LPS-induced COX-2 *in vivo* and vitro. J. Henan Normal Univ. Sci. Ed. 45 (5), 49–52. 10.16366/j.cnki.1000-2367.2017.05.009

[B32] Kang J. Zhang Y. Cao X. Fan J. Li G. Wang Q. (2011). Lycorine inhibits lipopolysaccharide-induced iNOS and COX-2 up-regulation in RAW264.7 cells through suppressing P38 and STATs activation and increases the survival rate of mice after LPS challenge. Int. Immunopharmacol. 12 (1), 249–256. 10.1016/j.intimp.2011.11.018 22155741

[B33] Karolina Ś. Elżbieta S. Jan O. Jan O. Joanna K. O. (2016). A micelle-mediated extraction as a new method of obtaining the infusion of Bidens tripartita. Acta biochim. Pol. 63 (3), 543–548. 10.18388/abp.2015_1223 27231727

[B34] Lin Q. (2019). ChlorogenicAcid inhibits TLR4/NF-κB signaling pathway and induced apoptosis in macrophage-derived foam cells. J. North Pharm. 16 (8), 123–126.

[B35] Lan C. Meng L. Chen C. Lan L. Wang X. Chang S. (2021). Chlorogenic acid inhibits glycolysis and fatty acid metabolism and regulates M1 macrophage polarization. Chin. J. Immunol. 37 (20), 2440–2444.

[B36] Lee T. K. Kang I. J. Kim B. Sim H. J. Kim D. W. Ahn J. H. (2020). Experimental pretreatment with chlorogenic acid prevents transient ischemia-induced cognitive decline and neuronal damage in the Hippocampus through anti-oxidative and anti-inflammatory effects. Molecules 25 (16), 3578. 10.3390/molecules25163578 32781658 PMC7463954

[B37] Lee Y. Bae C. S. Ahn T. (2022). Chlorogenic acid attenuates pro-inflammatory response in the blood of streptozotocin-induced diabetic rats. Laboratory Animal Res. 38 (1), 37. 10.1186/s42826-022-00148-x PMC9719206 36461118

[B38] Li H. Xu J. Hu J. Hu Q. Fang X. Sun Z. (2022). Sustained release of chlorogenic acid-loaded nanomicelles alleviates bone loss in mouse periodontitis. Biomaterials Sci. 10, 5583–5595. 10.1039/d2bm01099b 35975567

[B39] Li K. Feng Z. Wang L. Ma X. Wang L. Liu K. (2023). Chlorogenic acid alleviates hepatic ischemia-reperfusion injury by inhibiting oxidative stress, inflammation, and mitochondria-mediated apoptosis *in vivo* and *in vitro* . Inflammation 46, 1061–1076. 10.1007/s10753-023-01792-8 36856879 PMC10188389

[B40] Li X. Zhu S. Yin P. Zhang S. Xu J. Zhang Q. (2021). Combination immunotherapy of chlorogenic acid liposomes modified with sialic acid and PD-1 blockers effectively enhances the antitumor immune response and therapeutic effects. Drug Deliv. 28 (1), 1849–1860. 10.1080/10717544.2021.1971797 34515617 PMC8439241

[B41] Liang N. Kitts D. D. (2018). Chlorogenic acid (CGA) isomers alleviate interleukin 8 (IL-8) production in caco-2 cells by decreasing phosphorylation of p38 and increasing cell integrity. Int. J. Mol. Sci. 19 (12), 3873. 10.3390/ijms19123873 30518116 PMC6320834

[B42] Liu D. Wang H. Zhang Y. Zhang Z. (2020). Protective effects of chlorogenic acid on cerebral ischemia/reperfusion injury rats by regulating oxidative stress-related Nrf2 pathway. Drug Des. Dev. Ther. 14, 51–60. 10.2147/DDDT.S228751 PMC6954849 32021091

[B43] Liu H. Yao Y. (2005). Advances in cross talk of cellular signalling pathways associated with inflammatory response. Chin. J. Pathophysiol. 21 (8), 1607–1613.

[B44] Liu J. Cai Y. Liu F. Chen B. Gu Z. Chen S. (2020a). Biological function of chlorogenic acid and its application in animal husbandry. China feed. 651 (7), 9–12.

[B45] Liu J. Zhang Z. Lin J. Li Y. Luo T. Chen Q. (2020b). Animal experimental study on the treatment of oral ulcer by chlorogenic acid. China Med. Pharm. 10 (23), 41–44.

[B46] Liu Y. Guo H. q. Yang L. Cheng Y. Shui X. Yang L. (2019). Study on anti-inflammatory activities *in vitro* and *in vivo* and relation of a fingerprint with pharmacodynamics of Notopterygii Rhizoma et Radix from three commercial specifications. Chin. Traditional Herb. Drugs 50 (24), 6052–6058.

[B47] Liu Y. Wang F. Li Z. Mu Y. Yong V. W. Xue M. (2022). Identification, screening, and comprehensive evaluation of novel DPP-IV inhibitory peptides from the Tilapia skin gelatin hydrolysate produced using ginger protease. Biomolecules 12 (8), 1866. 10.3390/biom12121866 36551294 PMC9775409

[B48] Masato K. Tatsuya M. Takayuki H. Yukiko N. Satoshi K. Takeshi M. (2019). Coffee with a high content of chlorogenic acids and low content of hydroxyhydroquinone improves postprandial endothelial dysfunction in patients with borderline and stage 1 hypertension. Eur. J. Nutr. 58 (3), 989–996. 10.1007/s00394-018-1611-7 29330659 PMC6499758

[B49] Matthias G. Alexey K. Michael K. (2009). Targeting innate immunity protein kinase signalling in inflammation. Nat. Rev. Drug Discov. 8 (6), 480–499. 10.1038/nrd2829 19483709

[B50] Meinhart A. D. Damin F. M. Caldeirão L. Filho M. d. J. Silva L. C. d. Constant L. d. S. (2019). Study of new sources of six chlorogenic acids and caffeic acid. J. Food Compos. Analysis 82 (C), 103244. 10.1016/j.jfca.2019.103244

[B51] Munawaroh F. Arfian N. Saputri L. A. A. W. S. Kencana S. M. S. Sar D. C. R. (2023). Chlorogenic acid may improve memory function and decrease inflamation of frontal lobe in diabetic rat. Med. J. Malays. 78 (4), 476–483. 37518915

[B52] Niu P. Zhou X. Bai M. (2022). Influence of chlorogenic acid from lycium barbarum on cardiac fibroblast fibrosis induced by transforming. Growth factor-β1. Food Sci. 43 (7), 158–164.

[B53] Patrícia d. D. L. Naiara d. A. Ferreira d. A. N. Maquilon M. O. L. Talita d. O. S. J. K. Gomes M. E. C. M. (2022). COX/iNOS dependence for angiotensin-II-induced endothelial dysfunction. Peptides 157, 170863. 10.1016/j.peptides.2022.170863 36028074

[B54] Qian C. Liu J. Cao X. (2014). Innate signaling in the inflammatory immune disorders. Cytokine & growth factor Rev. 25 (6), 731–738. 10.1016/j.cytogfr.2014.06.003 25007741

[B55] Sabna K. Mubarak A. H. Kamal Y. Nair A. B. Yt K. (2022). Progress in polymeric micelles for drug delivery applications. Pharmaceutics 14 (8), 1636. 10.3390/pharmaceutics14081636 36015262 PMC9412594

[B56] Shiva R. Parisa S. Firouz G. Roya N. (2022). miR-23b/TAB3/NF-κB/p53 axis is involved in hippocampus injury induced by cerebral ischemia-reperfusion in rats: The protective effect of chlorogenic acid. BioFactors Oxf. Engl. 48 (4), 908–917. 10.1002/biof.1830 35201648

[B57] Simona T. Antonella A. Antonio M. Giuseppa C. Pasquale C. Roberta C. (2023). A nutraceutical containing chlorogenic acid and luteolin improves cardiometabolic parameters in subjects with pre-obesity: A 6-month randomized, double-blind, placebo-controlled study. Nutrients 15 (2), 462. 10.3390/nu15020462 36678333 PMC9862908

[B58] Song D. Zhang S. Song Z. Shi S. (2023). Research progress on the structural and functional comparison, structural modification of chlorogenic acid and its isomers and application in animals. Chin. J. Animal Sci. 59 (1), 10–19. 10.19556/j.0258-7033.20211213-03

[B59] Song Y. Wang H. Ni F. Wang X. Zhao Y. Huang W. (2015). Study on anti-inflammatory activities of phenolic acids from Lonicerae Japonicae Flos. Chin. Traditional Herb. Drugs 46 (4), 490–495.

[B60] Tamanna R. Kanta D. S. Ananya P. Das C. A. Malay D. Mandal S. M. (2022). Facile and green fabrication of highly competent surface-modified chlorogenic acid silver nanoparticles: Characterization and antioxidant and cancer chemopreventive potential. ACS omega 7 (51), 48018–48033. 10.1021/acsomega.2c05989 36591115 PMC9798512

[B61] Tan S. Yan F. Li Q. Liang Y. Yu J. Li Z. (2020). Chlorogenic acid promotes autophagy and alleviates Salmonella typhimurium infection through the lncRNAGAS5/miR-23a/PTEN Axis and the p38 MAPK pathway, Frontiers in cell and developmental biology. 10.3389/fcell.2020.552020 PMC7682651 33240872

[B62] Tang Y. Cao L. Gan L. Xiao J. Liu Y. Daraqel B. (2022). Three-dimensional analysis of alveolar bone morphological characteristics in skeletal class II open bite malocclusion: A cone-beam computed tomography study. Prog. Anatomical Sci. 28 (1), 39–41. 10.3390/diagnostics13010039 PMC9818805 36611329

[B63] Tapan K. G. Ayan G. Tapan K. B. Subhasis M. (2016). Nanoliposome is a promising carrier of protein and peptide biomolecule for the treatment of cancer. Anti-cancer agents Med. Chem. 16 (7), 816–831. 10.2174/1871520616666151116121821 26567624

[B64] Tian L. Su C. Wang Q. Wu F. Bai R. Zhang H. (2019). Chlorogenic acid: A potent molecule that protects cardiomyocytes from TNF-α-induced injury via inhibiting NF-κB and JNK signals. J. Cell. Mol. Med. 23 (7), 4666–4678. 10.1111/jcmm.14351 31033175 PMC6584503

[B65] Wang D. Hou J. Wan J. Yang Y. Liu S. Li X. (2021). Dietary chlorogenic acid ameliorates oxidative stress and improves endothelial function in diabetic mice via Nrf2 activation. J. Int. Med. Res. 49 (1), 300060520985363. 10.1177/0300060520985363 33472479 PMC7829538

[B66] Wang D. Tian L. Lv H. Pang Z. Li D. Yao Z. (2020a). Chlorogenic acid prevents acute myocardial infarction in rats by reducing inflammatory damage and oxidative stress. Biomed. Pharmacother. 132, 110773. 10.1016/j.biopha.2020.110773 33022535

[B67] Wang H. Tian L. Han Y. Ma X. Hou Y. Bai G. (2022a). Mechanism assay of honeysuckle for heat-clearing based on metabolites and metabolomics. Metabolites 12 (2), 121. 10.3390/metabo12020121 35208196 PMC8874459

[B68] Wang J. Rao C. Dai B. Yu H. Wang Z. (2023a). The sound of silence: Cognitive biases and socioeconomic barriers leading to delayed diagnosis of metastatic cancer. Chin. J. General Pract. 21 (3), 405–406. 10.1016/j.jaclp.2023.02.004 37474244

[B69] Wang T. Yin L. Ma Z. Zhang Y. (2022b). Chlorogenic acid-loaded mesoporous silica nanoparticles modified with hexa-histidine peptides reduce skin allergies by capturing nickel. Molecules 27 (4), 1430. 10.3390/molecules27041430 35209219 PMC8876321

[B70] Wang W. Wen C. Guo Q. Duan Y. Li Y. He S. (2017b). Biological properties of chlorogenic acid and its mechanism of action. Chin. J. Animal Nutr. 29 (7), 2220–2227.

[B71] Wang X. Chen M. Xiao H. Liu Y. Wang R. Jiang L. (2023b). Causal relationships between interleukins, interferons and COVID-19 risk: A mendelian randomization study. Food Sci., 1–10. 10.1080/09603123.2023.2252461 37660260

[B72] Wang X. He P. Wang W. Zhao H. Yi S. Wang C. (2020b). Integrated profiling of fatty acids, sterols and phenolic compounds in tree and herbaceous peony seed oils: Marker screening for new resources of vegetable oil. Immunol. J. 36 (9), 770–776. 10.3390/foods9060770 PMC7353516 32545196

[B73] Wang Y. Zhang L. Chen Q. Li J. (2017a). Effect of chlorogenic acid on the secretion of inflammatory cytokines in human hepatic stellate cells induced by TGF-β1. Chin. Pharm. J. 52 (6), 452–456.

[B74] Wu L. Shi Z. Zhao P. Song W. Xu M. Zhu Z. (2022). Therapeutic effect of isochlorogenic acid on salpingitis in hy-line grey laying hens. China Poult. 44 (9), 47–52. 10.16372/j.issn.1004-6364.2022.09.008

[B75] Xian L. Jiang Y. Tian P. Liang L. (2019). Chlorogenic acid regulates lipopolysaccharide-induced apoptosis and inflammatory response of human retinal vascular endothelial cells. Chin. J. Immunol. 35 (2), 171–175+180.

[B76] Xiong W. Li X. Hu J. Fu J. Tu Z. Wang H. (2013). Preparation of chlorogenic acid nanoliposome and its quality evaluation. Food Res. Dev. 34 (19), 12–16.

[B77] Xu P. Xu X. Hanna F. Tetiana F. (2023). Anti-inflammatory effects of chlorogenic acid from *Taraxacum officinale* on LTA-stimulated bovine mammary epithelial cells via the TLR2/NF-κB pathway. PloS one 18 (3), e0282343. 10.1371/journal.pone.0282343 36947494 PMC10032541

[B78] Yao M. McClements D. J. Zhao F. Craig R. W. Xiao H. (2017). Controlling the gastrointestinal fate of nutraceutical and pharmaceutical-enriched lipid nanoparticles: From mixed micelles to chylomicrons. NanoImpact 5, 13–21. 10.1016/j.impact.2016.12.001

[B79] Yu P. Xiao W. Zhao L. (2018). Advances in the structure-activity relationships of chlorogenic acid derivatives. Chin. J. Med. Chem. 28 (2), 144–146. 10.1200/JOP.17.00084

[B80] Zang Y. Cao Y. Wu X. (2022). Coffee consumption and type 2 diabetes mellitus. Occup. Health 38 (3), 425–428+432.

[B81] Zhang S. Li H. Liu Q. Li X. Yang W. Zhou Y. (2023b). Eucommiae folium and active compounds protect against mitochondrial dysfunction-calcium overload in epileptic hippocampal neurons through the hypertrophic cardiomyopathy pathway. Neurochem. Res. 48 (9), 2674–2686. 10.1007/s11064-023-03937-5 37067737

[B82] Zhang W. Xu X. (2022). Effects of chlorogenic acid on renal inflammation and apoptosis in diabetes nephropathy rats based on TLR4/NF-κB signaling pathway. Mod. J. Integr. Traditional Chin. West. Med. 31 (19), 2629–2635.

[B83] Zhang Y. Zhu C. Zhao H. Sun Z. Wang X. (2023a). Anti-inflammatory effect of chlorogenic acid in Klebsiella pneumoniae-induced pneumonia by inactivating the p38MAPK pathway. Int. J. Med. Microbiol. 313 (2), 151576. 10.1016/j.ijmm.2023.151576 36812841

[B84] Zhao L. Zhang H. Xu M. Bao X. Ai X. Chen Y. (2021). Anti-inflammatory effect of stevia residue extract and its main components isochlorogenic acids. J. Chin. Inst. Food Sci. Technol. 21 (5), 117–124. 10.1186/s12876-021-01701-z

[B85] Zhao X. (2021). Study on the anti-inflammatory activity and preliminary mechanism of cryptochlorogenic acid form Mulberry leaf. China, Northeast Forestry University.

[B86] Zhao X. Yu L. Zhang S. Ping K. Ni H. Qin X. (2020). Cryptochlorogenic acid attenuates LPS-induced inflammatory response and oxidative stress via upregulation of the Nrf2/HO-1 signaling pathway in RAW 264.7 macrophages. Int. Immunopharmacol. 83 (C), 106436. 10.1016/j.intimp.2020.106436 32234671

[B87] Zheng Y. Li L. Chen B. Fang Y. Lin W. Zhang T. (2022). Chlorogenic acid exerts neuroprotective effect against hypoxia-ischemia brain injury in neonatal rats by activating Sirt1 to regulate the Nrf2-NF-κB signaling pathway. Cell. Commun. Signal. 20 (1), 84. 10.1186/s12964-022-00860-0 35689269 PMC9185968

[B88] Zhou Z. Wang D. Xu X. Dai J. Lao G. Zhang S. (2022). Myofibrillar protein-chlorogenic acid complexes ameliorate glucose metabolism via modulating gut microbiota in a type 2 diabetic rat model. Food Chem. 409, 135195. 10.1016/j.foodchem.2022.135195 36571901

[B89] Zhu C. Xu J. Wang X. Chen M. (2019). Improvement of chlorogenic acid for oxidative renal injury in diabetic rats through inhibiting endoplasmic reticulum stress. China Pharm. 22 (10), 1824–1828.

[B90] Zhu Z. Wang Q. Han J. Dou W. Jiang Z. Chen W. (2021). Promotion effect of chlorogenic acid on activation of mouse macrophages to type M2 and its mechanism. Guangxi Med. J. 43 (2), 200–204.

